# Tailor-Made Zinc-Finger Transcription Factors Activate *FLO11* Gene Expression with Phenotypic Consequences in the Yeast *Saccharomyces cerevisiae*

**DOI:** 10.1371/journal.pone.0000746

**Published:** 2007-08-15

**Authors:** Jia-Ching Shieh, Yu-Che Cheng, Mao-Chang Su, Michael Moore, Yen Choo, Aaron Klug

**Affiliations:** 1 Department of Biomedical Sciences, Chung Shan Medical University, Taichung, Taiwan; 2 Medical Research Council Laboratory of Molecular Biology, Cambridge, United Kingdom; 3 Department of Otorhinolaryngology–Head and Neck Surgery, Chung Shan Medical University Hospital, Taichung, Taiwan; Center for Genomic Regulation, Spain

## Abstract

Cys_2_His_2_ zinc fingers are eukaryotic DNA-binding motifs, capable of distinguishing different DNA sequences, and are suitable for engineering artificial transcription factors. In this work, we used the budding yeast *Saccharomyces cerevisiae* to study the ability of tailor-made zinc finger proteins to activate the expression of the *FLO11* gene, with phenotypic consequences. Two three-finger peptides were identified, recognizing sites from the 5′ UTR of the *FLO11* gene with nanomolar DNA-binding affinity. The three-finger domains and their combined six-finger motif, recognizing an 18-bp site, were fused to the activation domain of VP16 or VP64. These transcription factor constructs retained their DNA-binding ability, with the six-finger ones being the highest in affinity. However, when expressed in haploid yeast cells, only one three-finger recombinant transcription factor was able to activate the expression of *FLO11* efficiently. Unlike in the wild-type, cells with such transcriptional activation displayed invasive growth and biofilm formation, without any requirement for glucose depletion. The VP16 and VP64 domains appeared to act equally well in the activation of *FLO11* expression, with comparable effects in phenotypic alteration. We conclude that the functional activity of tailor-made transcription factors in cells is not easily predicted by the in vitro DNA-binding activity.

## Introduction

The Cys_2_His_2_ zinc finger (ZF) domain, initially discovered in TFIIIA from *Xenopus laevis* [1], [2], is the most common DNA-binding (DB) motif in eukaryotes, and the second most frequently-encoded protein domain in the human proteome, being found in approximately 2% of all our proteins [3]–[5]. Individual fingers of about 30 amino acids can bind RNA and protein, but they generally function by binding DNA. Each ZF module holds both an anti-parallel β-sheet and a recognition α-helix that interacts directly with ∼3 bps of DNA in the major groove. This structurally-simple ββα domain is stabilized by hydrophobic interactions and the chelation of single zinc ion between a pair of cysteines from the β-sheet and a pair of histidines from α-helix [6]. Significantly, Cys_2_His_2_ ZF domains are very suitable for the construction of artificial transcription factors (TFs) as they usually bind as covalent tandem repeats, allowing the recognition of extended asymmetrical DNA sequences. The modularity of the ZF proteins, both in structure and function, serves greater advantage in comparison with other types of DNA-binding domains (DBDs) that normally recognize DNA as dimers and use non-modular recognition domains [7], [8]. The Cys_2_His_2_ ZF of Zif268 has recently become known as a versatile scaffold for use in engineering novel DBDs with sequence-specificity [8]–[10] and has shown the ability to bind a variety of DNA sequences [11]–[13]. In addition, arrays of linked Zif268 finger-variants allow binding of longer asymmetric sequences [14], [15]. To date, customized TFs based on Zif268 have been constructed by a number of groups and shown to effectively activate or repress individual target genes in diverse biological systems [8], [10], [16]. Recently, we have shown that inhibition of HSV and HIV replication can be achieved by repression of HSV and HIV genes in their host cells, with tailor-made ZF TFs [17], [18]. The possibilities for therapeutic applications have been supported by reports that engineered ZF proteins can induce angiogenesis, suggesting a treatment for cardiovascular disease in a multi-cellular animal model [19], [20]. Beyond therapeutic applications, ZF proteins have also been used in the fields of drug discovery [21], stem cell research [22], [23], and gene targeting with sequence-specific integrases, nucleases, and methylases [17], [24]–[28].

In addition to developing methods of selection and applications in therapeutics, we are particularly interested in extending the use of ZF-based TFs to functional genomics. To gain insights on how the regulation of the gene expression is achieved by engineered TFs, such that altered gene expression leads to specific phenotypes for selection, we have taken advantage of the well-studied yeast *Saccharomyces cerevisiae*. This is a convenient model to study the behavior of tailor-made TFs to regulate the expression of genes with phenotypic consequences. We have chosen the *FLO11* (also known as *MUC1*) gene that is a downstream target on pathways of invasive growth in haploid yeast, because the pathways [29]–[31] and the gene itself are well characterized. *FLO11* is a adhesin-encoding gene whose product is a large, cell wall-associated, glycosylphosphatidylinositol (GPI)-anchored threonine/serine-rich protein, with structural similarity to mammalian mucins and yeast flocculins [32], [33]. *FLO11* is required for either pseudohyphal formation in diploid yeast upon nitrogen depletion [34] or invasive growth and cell-cell adhesion in haploid yeast when depleted of carbon source [31], [35]. The ability of haploid cells to invade and to initiate flocculation is achieved by overexpressing *FLO11* without the need for carbon source depletion [35]. To date, many transcriptional activators of *FLO11* have been shown to be needed for the induction of filamentous or invasive growth, through various signaling pathways, including Kss1 and cAMP-regulated as well as glucose repression cascades [30], [36]. These parallel pathways converge on the *FLO11* promoter, with at least four upstream activation sequences (UASs) and nine repression elements, which together span a minimum of 2.8 kb, in one of the largest promoters in *S. cerevisiae* genome [37]. Deletion of the repression region of the *FLO11* promoter appears to increase *FLO11* expression [38]. Intriguingly, *FLO11* promoter-dependent epigenetic variegation of *FLO11* expression has recently been revealed [39]. To add even more complexity, the presence of internal tandem repeats within the coding region of *FLO11* have been shown to maximize its expression by the THO complex at the level of transcriptional elongation [40]. Notably, invasive growth in haploid yeast, induced by transcriptional activation of *FLO11*, has been shown by overproduction of transcriptional regulators such as Msn1 and Mss11 [41] as well as Flo8 and Ste12 [37].

In this report, we show that three-finger (3F) peptides, selected by the bipartite approach of phage display [42], recognize two 9-bp sites from the 5′ un-translated region (5′ UTR) of the *FLO11* gene. The combined six-finger (6F) peptides (derived by linking the 3F peptides) recognize an 18-bp site with a 2-bp gap. Recombinant TFs (rTFs) were made by fusing these peptides to the activation domain (AD) VP16, from herpes simplex virus, or its derivative VP64. Despite finding several candidates that work in vitro, we found that only one peptide can competently activate the expression of *FLO11* in haploid yeast cells. The activation of *FLO11* expression is not dependent on carbon source depletion and can advance invasive growth of cells in semi-solid medium, biofilm formation of cells in liquid culture, and tighter adhesion of cells to polysterene. The inability of the rest of rTFs to efficiently activate *FLO11* expression is presumably due to exclusion from the binding site by endogenous factors binding to the site for the rTFs. Our results suggest that artificial TFs based on ZFs can be used as a general gene-switch tool for modulating the expression of specific genes that render specific selectable phenotypic alterations.

## Results

### Phage clones expressing zinc fingers are selected against pre-determined FLO11 DNA target sites

**Figure 1:**
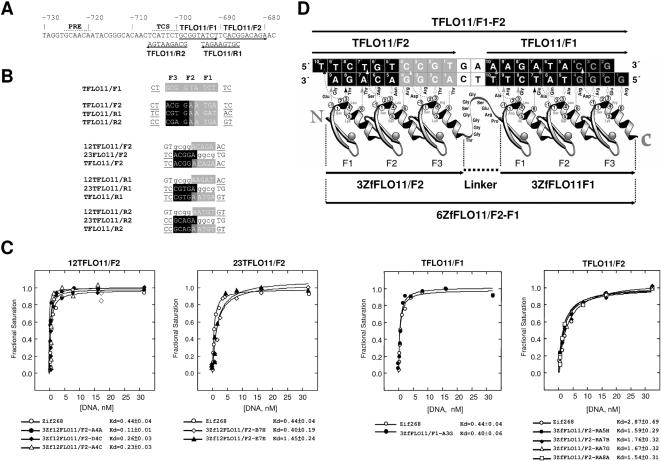
Binding of ZF domains to the *FLO11* gene of budding yeast *S. cerevisiae*. (A) 5′UTR sequences of *FLO11* used in identification of sequence-specific 3-finger DNA-binding motifs. Nucleotides are numbered relative to the translational start site. The 9-bp core sequences of the target sites TFLO11/F1, TFLO11/F2, TFLO11/R1 and TFLO11/R2 are shown with arrows indicating the direction of the sequence from 5′ to 3′. Sequences of Ste12 (PRE) , and Tec1 (TCS) binding sites are shown above. (B) DNA oligos used in the phage selection, including 2-bp flanking sequences on each side. The shaded areas represent the core bound sequences. Individual fingers bind triplet nucleotides, indicated by F1, F2, and F3, respectively. For direct selection of TFLO11/F1 from Lib12, the oligo CTGCGGTATCTTC is shown shaded light grey. Targets of TFLO11/F2, TFLO11/R1, and TFLO11/R2 were subjected to bipartite selection: targets were divided into two “half sites” that comprise a gcgg anchor (in lower case) and a 5-bp target (shaded grey or black). The targets were selected from two complementary libraries, Lib12 and Lib23, and recombined to bind a full 9 bp sequence, made from the two grey and black half-targets. The sequences flanking the core target sequences are underlined. (C) The apparent *K*_d_s of the individual clones bound to the respective 12TFLO11/F2 and 23TFLO11/F2 “half sites”, as well as the individual clones of TFLO11/F1 or TFLO11/F2 “full sites”. (D) Corresponding amino acid residues of 3ZfFLO11/F1 and 3ZfFLO11/F2 and their proposed mode of contact with DNA targets. The amino acids are shown with circles numbered relative to their helical positions. Due to a cross-strand interaction of amino acid 2 of finger 1, each of the 3F domains potentially binds to an extra base, making a 10-bp DNA site. The linker peptide between the two 3F domains is Thr-Gly-Gly-GlyGly-Ser-Gly-Gly-Ser-Glu-Arg-Pro. The amino acid residues with proposed contacts to DNA bases are shown with arrows. The shaded dotted arrows indicate that the contact is either stereochemically unfavorable (in black), favorable (in white), or neutral (in gray) according to “recognition-code” rules [43], [44].

To select phage clones expressing 3F peptides, a phage display approach was adopted. The Lib12 phage library was used directly to screen the 3F peptides against the target site TFLO11/F1 (Figure 1B). One phage clone from Lib12, binding to the 9 bp of TFLO11/F1, was thus obtained. Three paired targets, against 5 bp “half sites” from TFLO11/F2, TFLO11/R1, or TFLO11/R2 (Figure 1B), were selected in parallel using two complementary phage libraries, Lib12 and Lib23 (43). Thus, phage clones were obtained that bound to all the “half sites”: 12TFLO11/F2 and 23TFLO11/F2; 12TFLO11/R1 and 23TFLO11/R1; 12TFLO11/R2 and 23TFLO11/R2. All of the initially-selected clones were screened by phage ELISA to determine which ZFPs bound DNA with an affinity comparable to that of Zif268 to its cognate target.

To engineer clones capable of binding to their full-length 9 bp targets, those binding with paired overlapping 5 bp “half sites” were recombined according to the bipartite protocol, such that the recombinant clones would recognize the full-length targets (43). Four clones binding to the full-length target of TFLO11/F2 were identified after recombination from a mixture of phage clones recognising the respective 12TFLO11/F2 and 23TFLO11/F2 “half sites”. The apparent *K*_d_s of the individual clones are shown in Fig. 1C. The TFLO11/R1 and R2 clones gave no detectable binding after recombination, even though their “half site” modules bound DNA with an apparent affinity equal of better than Zif268 by phage ELISA (data not shown). Therefore only the four recombinant clones binding to TFLO11/F2 and the one clone binding to TFLO11/F1 were subjected to further analysis.

To determine the binding affinity of each selected or recombinant clone, the apparent *K*_d_ of the individual clones were assessed by phage ELISA. The recombinant clones, expressing 3F peptides, were tested for binding against serial dilutions of their target sites of either TFLO11/F1 or TFLO11/F2, and were found to display apparent *K*_d_ comparable to that of Zif268 to its cognate site, which is in the expected range of 10^−9^ to 10^−10^M (Figure 1C). These clones were then sequenced. The amino acid sequences, deduced from DNA sequences of the four clones 3ZfFLO11/F2, showed that they were identical. The amino acid sequence of the helical region from either 3ZfFLO11/F1 or 3ZfFLO11/F2 is shown in Fig. 1D. We note that the DNA-protein contacts do not entirely follow the principle of recognition-code stereochemistry [43], [44], even though they have survived a selection pressure for both affinity and specificity, by adding competitor DNA to the phage binding mixture (43).

### Tailor-made recombinant transcription factors derived from selected zinc fingers are capable of binding to DNA sites *in vitro*

To generate functional rTFs, the 3F peptides were fused to either VP16 or VP64 AD. These domains have been shown to be potent trancription activators, used in diverse biological systems including yeast *S. cerevisiae* [45], [46]. Four constructs were established, based on pcDNA3.1(−), that allowed expressing the rTFs of VP16-3ZfFLO11/F1, VP64-3ZfFLO11/F1, VP16-3ZfFLO11/F2, and VP64-3ZfFLO11/F2 in an *in vitro* T7 expression system. To increase the affinity and stability of the protein-DNA complex and to try to improve the *in vivo* function of the rTFs, a 6F rTF was also generated [14], [47]–[49]. The 6 fingers contained 3ZfFLO11/F2 in the N-terminus and 3ZfFLO11/F1 in the C-terminus, with a central linker containing TGGGGSGGSERP, able to span a gap of 2-bp between two 9-binding sites (3-bp gap, if the cross-strand position is counted as being in the gap; Moore, M., unpubl.; Figure 1D). This was then subcloned into pVP16 and pVP64 to allow expressing VP16-6ZfFLO11/F2-F1 and VP64-6ZfFLO11/F2-F1 *in vitro*.

**Figure 2:**
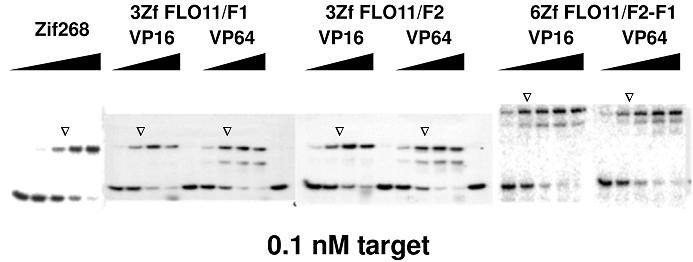
Zinc finger rTFs expressed from the pcDNA3.1(-)-based constructs retain their binding affinity when expressed as fusions with VP16 or VP64 activation domains, NLS and epitope tag. Gel-shift assays were carried out using the full target DNA site TFLO11/F2-F1, with rTFs produced from an *in vitro* transcription-translation system, using a 5-fold dilution series of protein. The binding of Zif268 with its cognate site (left) was used as a reference to determine the relative binding affinity of rTFs and TFLO11/F2-F1. To estimate Kd values, it was assumed that protein expression in each TNT reaction, and each subsequent dilution, was the same. Triangles indicate the concentration of rTFs where half the amount of target DNA bound with rTFs.

To assess whether fusing zinc fingers with ADs had any deleterious effect on DNA-binding affinity, gel-shift assays were conducted. TFLO11/F1-F2 DNA was tested against individual rTFs, produced from pV16-rTFs and pVP64-rTFs, in a transcription-translation extract. The results showed no difference in binding affinity of rTFs with either VP16 or VP64 (Figure 2), suggesting that the ADs in such constructs performed equally well, at least with respect to DNA-binding ability. Importantly, the binding affinity of 3ZfFLO11/F1 and 3ZfFLO11/F2 to the target sites appeared to be comparable to that of Zif268 to its cognate target site (Figure 2). This is consistent with results in the phage-ELISA where only the DBDs of 3F peptides were expressed. These observations indicated that additional domains fused to the C-terminus of ZFs, including nuclear localization signal and AD, did not change the binding affinity of the proteins tested. In addition, the binding affinity of the rTF with a 6F DBD was approximately 10-fold stronger to those of the rTFs with 3Fs (Figure 2). This is modest when compared with most of the previous observations that 6F proteins increase affinity for DNA from 20- to 6,000-fold, relative to 3F proteins [14], [15], [49]. Nonetheless, we decided to compare the relative functioning of these constructs *in vivo*.

### The induction of *FLO11* mRNA depends on the presence of recombinant transcription factors in *S. cerevisiae*

**Figure 3:**
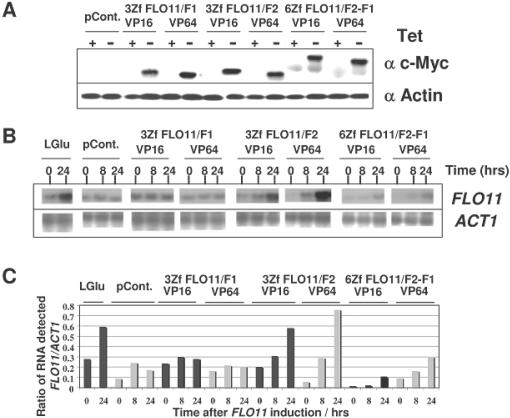
Zinc finger rTFs activate the expression of the *FLO11* gene in budding yeast. (A) DNAs capable of encoding rTFs were constructed into pCM185, a tetracycline-repressible yeast vector. Cells expressing rTFs, as indicated, were grown either in the presence or absence of 1 µg/ml tetracycline for 12 hrs, after which the cells were collected for Western blotting to detect the expression of rTFs. (B) The same cells were grown in the absence of tetracycline (inducing conditions) and collected at the times indicated, for Northern blotting to detect *FLO11.* The empty plasmid pCM185, designated as pCont., was used as a negative control. Cells with pCM185 growing in the absence of tetracycline and 0.05% glucose, indicated as LGlu, instead of 2% glucose were used to examine the expression of *FLO11* under derepressed conditions (C) The northern blots were quantitated using ImageQuant v1.2 and the ratios of induced FLO11, relative to *ACT1* control RNA, are plotted as a histogram.

To generate constructs capable of expressing functional rTFs in *S. cerevisiae*, the coding sequences of rTFs from pV16-rTFs and pVP64-rTFs were PCR amplified and cloned into pCM185 such that the expression of rTFs is tetracycline-repressible. The resulting six pCM185-rTF constructs allow expressing the rTFs of VP16-3ZfFLO11/F1, VP64-3ZfFLO11/F1, VP16-3ZfFLO11/F2, VP64-3ZfFLO11/F2, VP16-6ZfFLO11/F1-F2, and VP64-6ZfFLO11/F1-F2. These pCM185-rTFs constructs were introduced into wild type haploid yeast strain W303. To assess the expression of rTFs in yeast cells, the transformants with individual constructs were grown exponentially in selective medium, in non-induced conditions, followed by growing in either induced or non-induced conditions for various times. Samples were taken to measure the levels of rTFs expression by Western Blot analysis. The levels of rTFs began to accumulate after 3 hrs and reached a maximum within 12 hrs of induction (data not shown), consistent with the observed kinetics of induction in the system [50]. By contrast, no detectable rTFs were observed when cells were grown in non-induced conditions, as compared with those in induced conditions (Figure 3A). This regulatable mode of transcription control therefore provides a useful way to determine the dependency of gene expression by tailor-made rTFs targeting specific sequences of genes.

To test the dependency of the *FLO11* expression on rTFs, yeast cells carrying constructs of pCM185-rTFs were cultured exponentially in selective medium with glucose, in non-induced conditions. Samples were subsequently grown in induced or non-induced conditions for various times, and then the levels of *FLO11* mRNA examined by Northern Blot analysis. As shown in Figure 3B and C, while the *FLO11* mRNA only stayed at the basal level in cells carrying control pCM185, *FLO11* mRNA accumulated in cells expressing rTFs. This occurred in a time-dependent manner with the maximal level after 24 hrs of induction. Interestingly, such induction was particularly apparent in cells expressing rTFs with 3ZfFLO11/F2, although the induction could also be seen in cells expressing rTFs with 6ZfFLO11/F2-F1. Unexpectedly, the level of *FLO11* mRNA in cells expressing rTFs with 3ZfFLO11/F1 was similar to that in cells with the control, suggesting that this domain was not functioning *in vivo*.

It seemed that rTFs with VP64 activate the expression of *FLO11* better than those with VP16 (Fig. 3B, C). However, differences in activation between VP16 and VP64 were observed as being rather limited (data not shown). The potency of transcriptional activation between VP16 and VP64 is therefore by and large the same. These results indicated that the expression of *FLO11* mRNA was mediated by the induction of rTFs targeting the 5′UTR of *FLO11* gene. However, significant differences in transcriptional activation among rTFs were evident in that rTFs of 3ZfFLO11/F2 seemed to be the more potent transcriptional activators when compared with the rest of rTFs. Overall, this suggests that rTFs containing fingers targeting TFLO11/F1 were ineffective in activating the expression of *FLO11 in vivo*.

### The promotion of invasive growth in *S. cerevisiae* requires recombinant transcription factor-dependent expression of *FLO11* mRNA

**Figure 4:**
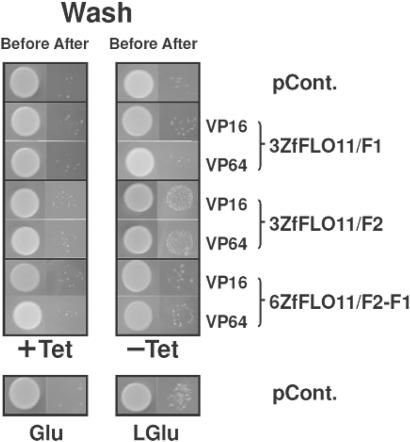
Zinc finger rTFs targeting the *FLO11* 5′UTR enhance invasive growth in budding yeast. Sequences encoding rTFs were subcloned into pCM185. Cells capable of expressing rTFs, as indicated, were grown in the presence of 1 µg/ml tetracycline, after which the cells were spotted onto semi-solid YEPD agar plates, using 10 µl of cells at an optical density at 600 nm (OD_600_) of 0.01. Plates were grown in induced (−Tet) or a non-induced (+Tet) conditions for two days at 30°C to form colonies. Colonies were then rinsed off the surface of the agar with a gentle stream of sterile dH_2_O. Invasive growth was shown by cells that remained inside the agar and that were able to repopulate the plate. The empty plasmid pCM185 (pCont.) was used as a negative control. Cells with pCM185 were grown and spotted on to semi-solid YEP agar plates with either 2% glucose (Glu) or 0.05% glucose (LGlu) in the absence of tetracycline to examine invasiveness under derepressed conditions.

Invasive growth in haploid *S. cerevisisae*, induced by transcriptional up-regulation of *FLO11*, has been shown by growing in medium depleted with glucose [35]. To investigate the enhancement of invasive growth in haploid *S. cerevisisae* being mediated by rTF-dependent expression of *FLO11* mRNA, haploid yeast cells carrying constructs of pCM185-rTFs were grown exponentially in selective medium with glucose, in either induced or non-induced conditions, and the improvement of invasiveness of cells was assessed by their ability to remain inside semi-solid YEPD agar, after being washed off from the surface of the plate. As shown in Figure 4, in non-induced conditions, only a restricted amount of new colonies formed from the remaining cells after washing. Under induced conditions, cells with control plasmid pCM185 alone showed limited invasive growth, as indicated in Figure 4. Similarly, cells expressing rTFs with either the 3ZfFLO11/F1 or 6ZfFLO11/F2-F1 DBDs exhibited only some degree of invasive growth, suggesting that those rTFs were not effective in up-regulating the expression of *FLO11* gene for a clear phenotypic alteration. By contrast, cells expressing rTFs with 3ZfFLO11/F2 became far more invasive, to an extent even greater than the derepressed conditions of low glucose (Figure 4). The three-finger 3ZfFLO11/F2, with either VP16 or VP64, was effective in triggering the expression of the *FLO11* gene to induce the phenotypic change.

### The enhancement of aggregation of S. *cerevisiae* cells requires recombinant transcription factor-dependent expression of *FLO11* mRNA

**Figure 5:**
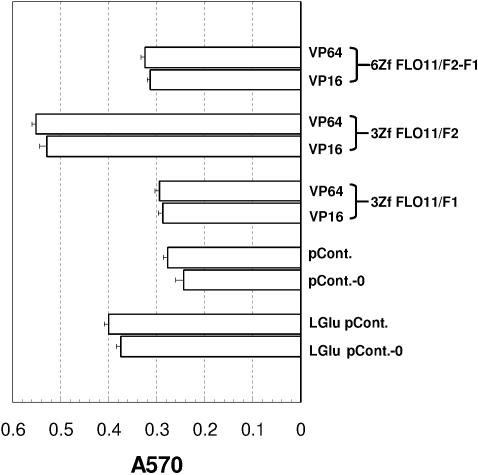
Adhesion assay to quantify the phenotypic effect of zinc finger rTFs targeting *FLO11* 5′UTR. Cells were assayed for the enhancement of biofilm formation by measuring the adhesion of budding yeast cells to polysterene plates; after washing to remove non-adhering cells, cells were stained with crystal violet, which was eluted and measured for OD at 570 nm. Cells expressing various rTFs are shown (in the absence of tetracycline; induced conditions). Cells carrying plasmid pCM185, grown with or without tetracycline, are designated as pCont.-0 and pCont., respectively. The fold-change in induction of adhesion was determined by dividing optical density at 570 nm of cells carrying rTFs with that of cells carrying pCM185 in the absence of tetracycline. Cells carrying pCM185, grown in YEP supplemented with 0.05% glucose, with or without tetracycline, subjected to adhesion assay are designated as LGlu pCont.-0 and LGlu pCont., respectively. Bars and error bars show means of three assays (each with three triplicates) and standard deviation. The Student t-test was use to determine the statistical significance between groups.

To investigate further the differences in potency among engineered TFs, via up-regulation of *FLO11*, a quantitative assay was adopted to measure the aggregation of yeast cells for biofilm formation [51], [52]. Haploid *S. cerevisisae* cells carrying constructs of pCM185-rTFs were grown exponentially in selective medium with glucose in non-induced conditions. These cells were then induced for 16 hrs prior to dispensing into wells of polysterene 96-microtiter plates, to allow cell attachment over a time-course. After staining with crystal violet for 30 mins, and after several washes, the plates were subjected to microscopic examination or determination of optical density at 570 nm. The cells with a better ability to form biofilms attach tighter to the wells of polystyrene plate, and thus stain with more crystal violet. The crystal violet on these cells, when eluted, displays a higher value of optical density. As shown in Figure 5, cells with control pCM185 plasmid were weakest in terms of their ability to adhere to polystyrene as compared with those containing rTFs under induced conditions (in the presence of glucose as carbon source). It appeared that cells expressing rTFs with 3ZfFLO11/F1 displayed only an insignificant ability to adhere than those of the control (pCont., p>0.1), with a fold-of-induction no more than 1.06 (Figure 5; Table S1; Table S2). Compared to those of the control (pCont.), cells with 6ZfFLO11/F2-F1 had only a fold-of-induction between 1.13 and 1.17 but enhanced adhesion with statistical significance (0.0051<p<0.0057). As before, the most prominent effect in enhanced adhesion was in cells expressing rTFs of 3ZfFLO11/F2, either with VP16 or VP64. These cells exhibited about 2-fold greater adhesion than the control (p<0.0001), suggesting that 3ZfFLO11/F2 is the most effective tailor-made rTF. Its effect on activation of *FLO11* expression leads to a clear aggregation phenotype that is independent of glucose depletion. Importantly, this aggregation phenotype appeared to be stronger than that of cells under low glucose derepressed conditions. Noticeably, cells expressing rTFs of the same DBD with AD VP64 appeared to induce biofilm formation more strongly than with VP16, though this effect was marginal and insignificant (0.1<p<0.53), considering the fact that the VP64 should be an AD with much stronger potency than VP16.

## Discussion

In this report, we studied engineered 3- and 6-finger rTFs targeting 9-bp and combined 18-bp sites of the 5′UTR of the *S. cerevisiae FLO11* gene, which is required for invasive growth and aggregation of haploid yeast cells. We showed that the engineered rTFs could activate the expression of the *FLO11* gene in *S. cerevisiae* haploid cells, bringing about a phenotypic alteration to promote invasiveness of growth and aggregation of cells.

One of the 3F peptides, 3ZfFLO11/F1, was successfully selected directly from the library Lib12, which contains variants of 3F peptides to target sites bearing the common anchor sequence of GCGG. Such selection remains a feasible approach so long as the target sequences chosen comprise the GCGG sequence; this has also been established by us in targeting the *GPD1* gene (manuscript in preparation) and for viral targets [17], [18]. By contrast, only one out of three clones that has been selected by phage display with the bipartite approach functioned here, even though the initial selected clones against each “half site” had *K*_d_ values equal to or greater than that of Zif268 to its cognate site. This is presumably due to loss of tighter DB affinity against the “full sites” due to incompatibilities within the recombined clones. The results indicate that at least several sites should be screened in parallel for a successful result with bipartite selection. Significantly, all the functional ZF DBDs obtained, either selected directly from Lib12 or via recombination after bipartite selection, have *K*_d_ values in the nanomolar range, similar to those of naturally existing TFs such as Sp1 and Zif268.

Notably, the rTFs with ZF DBDs and ADs, together with the short peptides of nuclear localization signal and epitope tag, maintained their DB affinity relative to proteins with isolated DBDs. It should also be emphasised that the ADs of either VP16 or VP64 played no apparent role in the affinity of DNA-binding of the rTFs. Similarly-structured engineered rTFs have also been shown to keep their DB affinity [17], [18], therefore this can be used as a general structure for engineering rTFs. Moreover, the rTFs carrying the 6F DB peptide (recognizing 18-bp with 2-bp gap) had an affinity greater than those carrying individual 3F peptides, although such increase in DB affinity is only moderate, at about 10-fold. The linker we adopted was a canonical type of linker with an insertion of 7 residues TG(GGGSGGS)ERP that should allow flexibility. This affinity increase is well below that reported by Kim and Pabo using similar linkers: they report an enhancement of binding affinity of 6,000-fold, albeit using a different method to measure the affinities [14]. The increase in affinity is also less than the one with a structural linker, which has been reported as being over 300-fold tighter [49]. This may well be the effect of linker peptide being perhaps not as flexible as expected, thus preventing the optimal proximity of the ZF resides to their contacting bases. It may also have arisen from the specific geometry of the interaction between ZF residues and the bases of the DNA-binding sites.

With respect to the ability of rTFs to activate the expression of *FLO11*, cells carrying rTFs with a DBD of 3F 3ZfFLO11/F2 (and an AD of either VP16 or VP64) appeared to be the most potent in activating expression of *FLO11*, resulting in a significantly altered phenotype. This new phenotype was easily distinguished from the control in the qualitative assay of invasive growth. In addition, such phenotypic change exhibited 2-fold enhancement of induction in a quantitative adhesion assay. The induction of adhesion could be maintained at the same level for up to 3 hrs in the presence of rTFs (data not shown), indicating that the maintenance of adhesion-induction is indeed dependent on the expression of the rTFs. By contrast, cells carrying rTFs with the DBD of 3F 3ZfFLO11/F1 (that was similar in DB affinity with 3ZfFLO11/F2, as determined by gel-shift assays) lacked any significant ability to activate the expression of *FLO11*. 3ZfFLO11/F1 behaved nearly the same as the control, with no apparent alteration in phenotype. Furthermore, the six finger 6ZfFLO11/F2-F1 that was approximately 10-fold tighter than 3ZfFLO11/F2 in DB affinity, also showed only a limited capacity in activating the expression of *FLO11*. The 6F rTF was in fact constructed to create a more potent transcriptional activator from the 3F components, due to a combination of a slower off-rate, an extended half-life, and a better binding affinity when bound with their target DNA sites [14], [53]. These effects should permit more efficient competition with the naturally occurring transcription factors, and yet 6ZfFLO11/F2-F1 works less well than 3ZfFLO11/F2. Since both 3ZfFLO11/F1 and 6ZfFLO11/F2-F1 target the common DNA site of TFLO11/F1, but are ineffective for *FLO11* transcriptional activation, there may be occlusion of the TFLO11/F1 site by endogenous TFs. Indeed, the TFLO11/F1 site is adjacent to the enhancer element of TEA/ATT with consensus sequence CATTCPy (TCS), that is bound by the endogenous TF Tec1 [54]. A recent study has shown that expression of filamentation genes such as *FLO11* is regulated by Tec1 binding on TCS, which is inhibited by Dig1 [55]. In non-filamentation-inducing conditions, binding of TCS is regulated by a complex of Tec1/Ste12/Dig1. This complex may be sufficient to exclude rTFs with the DBD of 3ZfFLO11/F1 from binding to TFLO11/F1, as it is adjacent to the TCS site. The rTFs with 6ZfFLO11/F2-F1, binding to TFLO11/F1-F2, may also be severely disrupted but still retain a slightly better ability than those with 3ZfFLO11/F1 alone, to activate the expression of *FLO11*. These results are different to those in a previous study, in which a 6F KOX repressor is able to exclude endogenous TFs from the HIV-1 5′ promoter [18]. Indeed, we cannot completely rule out the possibility that other sites in the genome bind with 3ZfFLO11/F1 or 6ZfFLO11/F2-F1 and thereby titer out their binding with TFLO11/F1 or TFLO11/F1-F2. However, it is also possible that the functional activity of rTFs in cells is determined by DNA accessibility in addition to affinity for the target site.

It appeared that cells expressing rTFs with VP64 activated the expression of *FLO11* in a level similar to those with VP16. Indeed, a quantitative assay for cell aggregation revealed that rTFs with VP64 were only 1.03- to 1.07-fold more effective in generating a signal than those with VP16 (0.1<p<0.53), which is statistically insignificant. Therefore while VP64 may be a better transcriptional activator than VP16, this results in a very limited difference in potency *in vivo*. The differences in fold-of-induction of phenotypic alteration should be in direct association with the up-regulation of the *FLO11*. The limited improvement in potency of VP64 over VP16 in rTFs is not as striking as in a previous report, in which an erbB-2 promoter-luciferase reporter system was targeted by transcriptional activators with ADs of either VP16 or VP64, resulting in a 5-fold increase in potency with VP64 [53]. To the best of our knowledge, no report has addressed the issue of the potency between VP16 and VP64 by comparing their ability to activate endogenous genes. Whether the reduced effect on transcriptional activation in targeting an endogenous gene, compared with a reporter system, is a general phenomenon or whether the effect is target gene-specific remains to be studied systematically. It is also of interest that although the expression of *FLO11* under derepressed conditions is comparable to rTFs with 3ZfFLO11/F2, the results from phenotypic assays indicated that rTFs with 3ZfFLO11/F2 enhance phenotypic alterations to a greater extent. This difference might be the result from other factors, in addition to *FLO11* induction under derepressed conditions, determining the outcoming phenotypes.

In this report, we have shown that a customized rTF can activate the expression of an endogenous gene in yeast cells, in a sequence-specific manner. Such regulations appear to mimic the control of gene expression by naturally existing TFs, but are amenable to artificial inducible regulation. The results complement other applications of zinc fingers for transcriptional therapy, as demonstrated previously in many other systems [8], [10], [16]–[18], [56]. Given that only ZF-based rTFs can act as either activators or repressors in the control of gene expression, they have certain advantages over other gene regulation technologies, such as RNAi, antisense, and ribozymes, which are limited to the inhibition of gene expression. Nonetheless, as with all other forms of gene therapy, the lack of effective delivery systems and the possibility of immunological response against the rTFs, may be the real hurdle for ZF-based transcriptional therapy. Nonetheless, ZF-based rTFs allow the regulation of expression of endogenous genes, resulting in phenotypic alterations. This opens up an opportunity in the use of ZF proteins in functional genomics. In certain cells, particularly those of lower eukaryotes such as yeast, screening specific variants of rTFs could readily be used for the selection of desired phenotypes, as has also been demonstrated recently [56], [57].

## Materials and Methods

### Library selection of zinc fingers by phage display

Four target sites (each of 9-bp) from the 5′UTR of *FLO11* were identified from the *Saccharomyces* Sequence Database , and were used for selection by phage display (Figure 1). These sites are all close to, or overlapping with, the cis-acting elements of the known UASs where the natural transcriptional regulators bind, to ensure the openness of the chromatin [58]. Target sites were made with 5′-end being biotyinylated and were subjected to selection using libraries containing randomized 3F peptides in a Fd-Tet-SN phage display system [59]. Zinc finger peptides (ZFPs) to bind to the target TFLO11/F1 (GCGGTATC) were screened directly from the phage library Lib12 with randomized peptides recognizing 5′-GCGGXXXXX-3′ sequences (Figure 1B), as described previously [13], [42]. ZFPs to bind to 12TFLO11/F2 and 23TFLO11/F2, 12TFLO11/R1 and 23TFLO11/R1, as well as 12TFLO11/R2 and 23TFLO11/R2, which are paired parts of the respective targets TFLO11/F2, TFLO11/R1, and TFLO11/R2 (Figure 1B), were selected in parallel using two complementary phage libraries, Lib12 and Lib23, giving two DBDs each of which recognizes a given 5-bp site [13], [42]. These products were recombined to produce single 3F peptides that recognize the full-length composite 9-bp sites of TFLO11/F2, TFLO11/R1, and TFLO11/R2, respectively.

### Evaluation of zinc finger binding affinity

The phage enzyme-linked immunosorbent assay (Phage-ELISA) was used to assess the binding affinity of the selected clones [60]. The apparent equilibrium dissociation constant (*K*_d_) value was used to determine the binding affinity of the selected clones. To establish *K*_d_ values, the phage-displayed peptides were assessed in ELISAs against serial dilutions of their 9-bp DNA target sites, with a range from 0.125 to 32 nM DNA. Wild type Zif268 (displayed on phage) was used as control against its cognate 9-bp binding site in these experiments (Figure 1C).

### Construction of a six-finger peptide

A 6F peptide was constructed by fusing two selected 3Fs whose target sites (TFLO11/F1 and TFLO11/F2) are 2 bps apart (3 bps if the cross-strand position is considered, see Figure 1D). The selected clone expressing 3F peptide that bound to TFLO11/F2 was placed on N-terminus of the full-length peptide and PCR amplified with primer 1 (GAGTTGCGTCT GACGCCGCCATGGCGGAGAGGCCCTACGCATGC) and primer 2 (GCCGCATCTTT TTGGTCCGTCCCCGCCCGTGTGTATCTTGGTATG). The clone expressing the 3F peptide that bound to TFLO11/F1 was placed on C-terminus of the full-length peptide and PCR amplified with primer 3 (TCAAGTTGCAGATCTGAGAGGCCCTACGCATGCCC TGTC) and primer 4 (CATTTAGGAATTCCGGGCCGCGTCCTTCTGTCTCAGATGGA TTTT) and Pfu polymerase, at the annealing temperature of 64°C. Equal amounts of N-terminus and C-terminus fragments from the PCR were subjected to *Bam*HI and *Bgl*II digestion in a volume of 20 µl for 2 hrs. The mix was supplemented with 3 µl each of 100 mM DTT and 10 mM ATP, 1 µl ligase, 0.5 µl each of *Bam*HI and *Bgl*II, and was incubated overnight at room temperature. To amplify the 6F fragment by PCR, 2 µl of the above ligation mix was used as a template with Pfu, primer 5 (CAGTTGCGTCTAGACGC CGCC), and primer 6 (CATTTAGGAATTCCGGGCCGC) at an annealing temperature of 64°C. The linker peptide between the two 3F fragments was TGGGGSGGSERP, which is an extended canonical type of linker with the insertion of GGGSGGS within the canonical linker sequence TGERP (Figure 1D).

### Cloning of zinc-finger constructs with activation domains

The selected or engineered clones encoding ZFPs were cloned into two modified pcDNA3.1(−) mammalian expression vectors (Invitrogen), pVP16 and pVP42. Both vectors were constructed such that sequences encoding a 7-aa nuclear localization sequence from simian virus 40 large T-antigen [61], an AD of either VP16 AD [45] or VP64 [46], and a C-terminus 10-aa sequence of the c-Myc 9E10 epitope [62], were cloned into the *Eco*RI and *Bam*HI restriction sites of pcDNA3.1(−). The coding regions of 3F peptides were amplified by PCR from the Fd-Tet-SN vector to have a 5′ *Xba*I restriction site, a Kozak sequence, and a 3′ *Eco*RI site. The 6F peptide construct contains a 5′ *Xba*I restriction site, a Kozak sequence, and a 3′ *Eco*RI site. The constructs were digested with *Xba*I and *Eco*RI and ligated into either pVP16 or pVP64 that had been digested previously with *Xba*I and *Eco*RI. The constructs capable of expressing rTFs *in vitro*, were assessed by the TNT T7 Quick Coupled Transcription/Translation System (Promega) with ^35^S-methione or Transcend™ Non-Radioactive Translation Detection System (Promega).

### Gel-shift assay

The target site used was a 31-bp oligonucleotide containing the suitable target sequence of 20-bp TFLO11/F1-F2, spanning TFLO11/F1 and TFLO11/F2. The oligonucleotides of the two complementary target sites were synthesized, annealed, and end-labeled with [^32^P]dATP. ZFPs were generated *in vitro* by the use of the TNT T7 Quick coupled Transcription/Translation System (Promega) and were subjected to gel-shift assays by the methods described previously [49]. *K*_d_ values and relative binding affinities were estimated by using a 5-fold dilution series of protein, from the in vitro transcription-translation system. It was assumed that protein expression in each TNT reaction, and thus active protein concentration in each subsequent dilution, was the same. An experiment was performed using the 3F Zif268 peptide against its 9-bp binding site as a calibration reference, based on the fact that Zif268 binds with a *K*_d_ of approximately 2 nM (51).

### Construction of yeast rTFs-expressing plasmids

The rTFs encoding ZFPs and AD of either VP16 or VP64 were cloned by PCR amplification from plasmids derived from pVP16 and pVP64. The 5′ oligonuclotide primer ZfNOT1N (GCCC*GCGGCCGC*AGCGCCGCCATGGCGGAAGAG) and the 3′ oligonucleotide primer ZfNOT1C (GGCC*GCGGCCGC*GTACAGATCTTCTTCAGAAAT) incorporate a *NOT*I site (shown italicized) and hybridize to sequences 5′ to the ZF and 3′ to the c-Myc 9E10 epitope. PCR-generated products were cleaved with *NOT*I and cloned into *NOT*I-linearized pCM185, downstream of a CYC1 promoter/TetO operator [50], to form pCM185-rTFs. Expression of these rTFs in *S. cerevisiae* was assessed by Western blot analysis.

### DNA sequencing

The coding sequences of individual clones expressing ZFPs were amplified by PCR from 1 µl of phage supernatant with two external primers, fdseq1 (GAATTTTCTGTATGAGG) and gIIIlead (GCAATTCCTTTAGTTGTTCC), complementary to phage sequence. These PCR products were sequenced with either fdseq1 or gIIIlead primers, by the Thermo Sequenase Cycle Sequencing kit (Amersham Biosciences). Dideoxy sequencing with T7 primer for pcDNA3.1(−) or with a specific primer for pCM185 was conducted using the T7 Sequencing kit (Amersham Biosciences), for individual constructs expressing rTFs.

### Yeast transformation

A haploid strain of wild type *S. cerevisiae* W303 (*Mat*a *ade2-1 his3-11,15 leu2-3,112 trp1-1 ura3-1 can1-100*) bearing the *trp1-1* marker was used to transform pCM185-rTFs as described previously [63]. The transformed cells were grown on agar plates of synthetic minimal medium containing 2% glucose and the required auxotrophic supplements, without tryptophan, at 30°C.

### Western blot analysis

To detect expression of the rTFs, *S. cerevisiae* cells with pCM185-rTFs were grown in synthetic minimal medium containing 2% glucose and the required auxotrophic supplements, without tryptophan, at 30°C, to retain pCM185-rTFs. To assess the expression of rTFs from the tetracycline-repressible promoter, cells were grown overnight in the presence of 1 µg/ml tetracycline (SIGMA), then diluted into fresh medium and grown exponentially. The cultures were then grown with (non-induction) or without (induction) 1 µg/ml tetracycline. Total protein was extracted from the cultured cells by the methods described previously [64]. The proteins were resolved by SDS-PAGE and transferred electrophoretically to nitrocellulose membranes. The membranes were probed with monoclonal antibody to c-Myc (9E10, Santa Cruz Biotechnology). Detection was conducted using a peroxidase-conjugated anti-mouse IgG (CHEMICON International). The signal was visualized by chemilluminescence (ECL, Amersham Biosciences) according to the manufacturer's instructions.

### Northern blot analysis

To isolate RNA, *S. cerevisiae* cells with pCM185-rTFs were grown in conditions as described in the previous section. Total RNA was extracted from the cultured cells and 10 µg samples, denatured with glyoxal, were resolved by 1.2% agarose gel electrophoresis before transferring to nitrocellulose for hybridization with ^32^P-labelled DNA probe as previously described [65]. To detect *FLO11* transcript, a probe consisting of internal fragments of the gene was amplified by PCR with primers of PFLO11CF (CTAGTTCCAAGAGGATCC) and PFLO11CR (CATCAAGCTCTACTACTACTG), complementary to *FLO11* sequence. Following autoradiography, appropriate exposures were used for analysis. The Northern blots were quantitated using ImageQuant v1.2 and the ratios of induced *FLO11*, relative to *ACT1* internal control RNA were thus obtained.

### Invasive growth assay

Cells carrying pCM-185-rTFs were grown overnight with 1 µg/ml tetracycline (SIGMA), then diluted into fresh medium and grown exponentially. The cultures were then grown with or without tetracycline in liquid synthetic minimal medium containing 2% glucose and the required supplements at 30°C and the morphology of cells were examined with a ZEISS AXioskop2 microscope at 400× agnification. The same cultures were spotted onto semi-solid YEPD agar plates, using 10 µl of cells at an optical density at 600 nm (OD_600_) of 0.01. Plates were grown in induced (−Tet) or a non-induced (+Tet) conditions for two days at 30°C to form colonies. Colonies were then rinsed off the surface of the agar with a gentle stream of sterile dH_2_O. Invasive growth was shown by cells that remained inside the agar and that were able to repopulate the plate.

### Adhesion assay

The adhesion of *S. cerevisiae* cells to the surface of polystyrene was determined quantitatively using crystal violet (SIGMA), essentially as described previously [51], [52]. Briefly, cells carrying pCM-185-rTFs were cultured in synthetic minimal medium containing 2% glucose and the required supplements at 30°C with 1 µg/ml tetracycline (SIGMA). Cells were then diluted into fresh medium and grown exponentially. The cultures were then grown with or without tetracycline in liquid synthetic minimal medium containing 2% glucose and the required supplements at 30°C and harvested at an optical density at 600 nm (OD_600_) of 0.5 to 1.0. Cells were then washed once in sterile dH_2_O, resuspended to 1.0 OD_600_ in liquid synthetic minimal medium containing 2% glucose and the required supplements, with or without tetracycline. A total of 100 µl cell suspension was transferred into wells of a 96-well polystyrene plate (Falcon Microtest flat bottom plate, 35-1172). After incubation for the required time at 30°C, an equal volume of 100 µl solution of 1% (w/v) crystal violet was added to the wells and left for 30 mins. The wells were washed three times with dH_2_O, eluted with 95% ethanol, and optical density was determined at 570 nm.

## Supporting Information

Table S1 (0.02 MB XLS)

Table S2 (0.02 MB XLS)
